# Comparison of ultrastructure, pollen tube growth pattern and starch content in developing and abortive ovaries during the progamic phase in hazel

**DOI:** 10.3389/fpls.2014.00528

**Published:** 2014-10-06

**Authors:** Jianfeng Liu, Huidi Zhang, Yunqing Cheng, Ju Wang, Yixin Zhao, Wanting Geng

**Affiliations:** Department of Life Science, College of Life Sciences, Jilin Normal UniversitySiping, China

**Keywords:** hazelnut, abortive ovary, pistillate inflorescence, fruit cluster, delayed fertilization

## Abstract

**HIGHLIGHTS**

- In an abortive ovary of hazel, an integument seldom differentiated and a mature embryo sac never developed.
- In an abortive ovary of hazel, pollen tube growth was arrested at the style base about 40 days after blooming. Thus, fertilization of the ovule was precluded.
- Ovary abortion in the four hybrid cultivars was indicated to be associated with insufficient resource availability to support fruit set by all flowers, whereas ovary abortion in C. heterophylla was at least partly determined by pollen availability.

In Northeast China, a high frequency of ovary abortion contributes to serious losses in yield of hazelnut. The development of pistillate inflorescences and fruit clusters of four large-fruited hybrid hazel cultivars and the small-fruited *Corylus heterophylla* were used to study ovary abortion and its possible causes during the progamic phase in hazel. The average number of pistillate (ANP) flowers per inflorescence and average number of fruit (ANF) per cluster of four hybrid hazel cultivars were 7.6–8.5 and 2.4–3.0 respectively; in *C. heterophylla*, its ANP and ANF was 5.8–6.2 and 3.5, respectively. The total drop varied from 50 to 67%. Ovary abortion in hazel initiated from about 30 days after blooming. The percentage of abortive ovaries (PAO) in the four hybrid hazel cultivars ranged from 63 to 72%, and was significantly higher than that of *C. heterophylla* (29–42%). Only the abortive ovary ratio of *C. heterophylla* was significantly reduced after artificial pollination. Fruit number per cluster was positively and negatively correlated with yield and nut mass, respectively. In abortive ovaries, the diameter remained less than 2 mm during the entire fruit development, an integument seldom differentiated and a mature embryo sac never developed. In addition, pollen tube growth was arrested at the style base about 40 days after blooming. Thus, fertilization of the ovule was precluded. Compared with abortive ovary, starch content in developing ovary of four hybrid hazel cultivars and *C. heterophylla* were significantly higher. This study suggests that abortive ovary was incapable to finish fertilization process due to the absence of mature embryo sac and arrested pollen tubes, and this is likely associate with insufficient resource availability to support fruit set by all flowers in four hybrid hazel cultivars, whereas ovary abortion in *C. heterophylla* is at least partly determined by pollen availability.

## Introduction

Hazel (*Corylus* spp.) is the most widely distributed and economically important member of the Betulaceae family in China. The hazel forest area in China exceeds one million hectares, which is much higher than all other hazelnut-producing countries in the world. The northeast is the traditional hazelnut production region in China. Development of the hazelnut industry plays an important role in increasing farmers' income, especially in mountainous areas (Liu et al., 2012). Compared with yields of 600–1000 kg ha^−1^ in Turkey, 2000–3000 kg ha^−1^ in Italy and 1700–2500 kg ha^−1^ in the United States (Aydinoglu, 2013), yields in China are less than 450 kg ha^−1^. Thus, investigating the factors responsible for the low yields and applying measures to enhance yields are important for development of the hazelnut industry in China.

Two *Corylus* L. species are indigenous to Europe. The European hazel, *C. avellana* L., is widely distributed and the Turkish hazel, *C. colurna* L., is restricted to the Balkans, Romania and northern Turkey (Thompson et al., 1996). European hazel was introduced to northeast China in the twentieth century. However, extremely low winter temperatures in Northeast China present an insurmountable barrier to cultivation of hazel species, which originate from temperate areas. *Corylus heterophylla* Fisch. ex Besser and its hybrids with European hazel (hybrid hazel; *C. heterophylla* × *C. avellana*) are the most important *Corylus* germplasm in China. The yields of hybrid cultivars account for more than 90% of hazelnut production in China. In addition, their excellent cold resistance characteristics ensure their dominance of hazelnut production in China in the short term.

Normal pollination and fertilization is a prerequisite for development of full-sized fruit in hazel. The fact that all pollinated fruits do not produce an edible kernel is a common phenomenon (Akiko and Hiroshi, 2006). Abortive ovaries of hazel flowers are incapable of developing into fruits and are destined to drop or wither. Ovary abortion varies greatly with year, cultivar, individual tree, branch, and shoot, and even among and within inflorescences (Fabbri et al., 2004; Martin and Sibbett, 2005). Ovary abortion of olive (*Olea europaea* L.) occurs early in flower development, mostly 30–40 days before blooming (Cuevas et al., 1999; Reale et al., 2006). Hazel species show unique delayed fertilization characteristics and ovary abortion is initiated several months after pollination (Beyhan and Marangoz, 2007). The average number of functional ovaries in one hazel inflorescence is 40–60% for the Tombul cultivar and 40–55% for the Palaz cultivar (Germain, 1994; Beyhan and Marangoz, 2007). Beyhan and Odabaş (1995) reported that 45–60% of the ovaries of hazel are abortive. Thus, only a small percentage of pollinated pistils of hazel develop into normal edible fruit (Thompson, 1967; Beyhan and Marangoz, 2007). A high frequency of ovary abortion in hybrid hazel and *C. heterophylla* has been observed in China in recent years, causing serious yield losses. However, the characteristics of hazel ovary abortion and the possible causal factors remain unknown. Therefore, the objective of this study was to characterize and investigate the possible causes of ovary abortion in hazel, so as to provide a scientific basis for development of strategies to reduce or overcome the yield losses resulting from ovary abortion.

## Materials and methods

### Study site and plant material

The trial was conducted at two towns of Siping City (Jilin province, China), including Shanmen (3 km southeast of Siping) and Yehe (30 km southeast of Siping). The two experiment zones are located in the North Temperate Zone and have a continental monsoon climate. The elevation varies from 110 to 240 m. The climate conditions of the two locations were very close. The annual average temperature and rainfall was 5.9°C and 572.8 mm, respectively. The average frost-free period is 142 days, and May to September is major growth seasons for crops. Both of them belong to dark-brown soil type, and their pH varies from 5.4 to 6.6. In Shanmen, 20-year-old trees of four hybrid hazel cultivars (“Bokehong,” “Dawei,” “Jinling,” and “Yuzhui”) were used as study materials. The spacing within and between rows was 3 and 4 m, respectively, and the tree height ranged from 2.5 to 3.0 m. In Yehe, 8-year-old *C. heterophylla* trees were used as study materials. The spacing within and between rows was 2 and 3 m, respectively, and the tree height ranged from 1.0 to 1.5 m. Female inflorescences and young fruits were sampled every 1–5 days from mid-April to late August in 2012 and 2013. The samples destined for histological examination or fluorescence microscopy observation were fixed in FAA solution (70% ethanol: glacial acetic acid: formalin, 18:1:1 [v/v/v]) for 3 days, then stored in 70% alcohol at 4°C.

### Measurement of development characteristics of pistillate flowers and fruit cluster

In both 2012 and 2013, more than 300 pistillate inflorescences from each of five germplasms were randomly sampled during the peak flowering period (mid-April). Every pistillate inflorescence was dissected with a scalpel and forceps, and the number of pistillate flowers per inflorescence and the percentage of pistillate flowers per inflorescence were recorded.

On April 1st of both years, pollen was collected from the hazel orchards and air-dried using the method described by Liu et al. (2012). After mixing equal amounts of pollen from each individual and pollen germination test on stigma of cultivar Dawei, artificial pollination was carried out in the peak flowering period (mid-April). Results of pollen germination test indicated that more than 75% pollen grains could germinate, while the germination ratio of fresh mixed pollen on fresh stigma of cultivar Dawei was about 86%. Twelve trees consistent in height and growth were selected for each hybrid hazel cultivar and *C. heterophylla*, and more than 300 pistillate inflorescences of each individual were artificially pollinated with brush and marked with tags. Concurrently, more than 300 open-pollinated pistillate inflorescences were randomly selected and marked with tags (the control). The tagged pistillate inflorescences and fruit clusters were monitored every week to determine the frequency of abscission from pollination to fruit harvest. On August 20th of both years, all remaining and tagged fruit clusters were harvested. Average number of fruit (ANF) per cluster, average number of abortive ovary (ANAO) per cluster, and percentage of abortive ovaries (PAO) per cluster were calculated. Fruit set as percentage of total number of pistillate flowers per plant (FS) was calculated using the following formula: *FS* = 100% × ∑ (fruit number after pistillate inflorescences tagged)/(number of tagged pistillate inflorescences × number of pistillate flowers per inflorescence). After dissection of the fresh nut, the shell mass, kernel mass and total nut mass were measured without drying. The kernel ratio was calculated with the following formula: kernel ratio = kernel mass × 100%/total nut mass.

### Observation of pollen tube growth, and ovary and ovule development in developing and abortive fruits

According to our previous histological examination, fertilization is completed about 60 days after blooming in hazel (Liu et al., 2012). In order to examine the relationship between pollen tube growth and ovary abortion, young pistillate inflorescences or fruit clusters of the hybrid hazel cultivar “Dawei” were collected to observe pollen tube growth and the anatomical structure of the ovary from 1 to 60 days after blooming. Pollen tubes in pistillate inflorescences or young fruit were stained with aniline blue (0.1 g aniline blue and 0.071 g K_3_PO_4_ per 100 ml distilled water), after bare-handed sectioned or squashing preparation, the stained specimens were to observed with a DM AE31EF-INV-5000C fluorescent microscope (Mike Audi Industrial Group Co., Ltd., China) with a UV filter set using the method of Liu et al. (2013) with minor modifications, and these modifications included samples softened in 1.0 M boiling NaOH solution for 10 min and neutralized in 0.1 M acetic acid solution during pretreatment stage.

Paraffin section was carried out in order to compare the morphological difference between developing and abortive ovaries, and the protocol of dehydration, staining, paraffin embedding and section of pistillate inflorescences or young fruit followed the method of Liu et al. (2012) with minor modifications in the pretreatment stage as described above.

### Starch assays in developing and abortive ovaries

In 2013 and 2014, pistillate inflorescences or young fruit cluster of four hybrid hazel cultivars and *C. heterophylla* were collected on 30, 40, and 50 days after blooming in the same orchard mentioned above. After fetching samples to laboratory, developing and abortive ovaries were separated and stored in liquid nitrogen. About 0.1 g sample was homogenized in a liquid N_2_ precooled mortar to measure starch content by using a pestle. The sample pretreatment and analysis followed the method described by Castro and Clément (2007).

### Statistical analysis

Statistical analysis was carried out with ANOVA process of SAS version 8.01 (SAS Institute, Inc., Cary, NC, USA). Means were compared using LSD (least significant difference) *t*-test at the 5% level of significance. Values expressed as a percentage were arcsin-transformed before analysis.

## Results

### Number of pistillate flowers per inflorescence

The percentage and mean number of pistillate flowers per inflorescence for each hybrid hazel cultivar and *C. heterophylla* are summarized in Table 1. The number of pistillate flowers per inflorescence ranged from four to 16 for the four hybrid hazel cultivars and from two to 16 for *C. heterophylla*. The number of pistillate flowers per inflorescence did not show a uniform and random distribution or a normal distribution. Instead, certain numbers of pistillate flowers per inflorescence showed a high frequency. For the hybrid hazel cultivars, six, eight, and 10 pistillate flowers per inflorescence were the most frequent numbers and their sum accounted for more than 60% of the total number of inflorescences. The most frequent numbers of pistillate flowers per inflorescence for *C. heterophylla* were four or six, which together made up more than 40% of the total number of inflorescences. The average number of pistillate (ANP) flowers per inflorescence ranged from 5.8 (in *C. heterophylla*) to 8.5 (in “Dawei” and “Bokehong”). The mean value for *C. heterophylla* was significantly lower than those of the four hybrid hazel cultivars.

**Table 1:** **Number of pistillate flowers per inflorescence in four hybrid hazel cultivars and *C. heterophylla***.

**Year**	**Cultivar/species**	**Number of pistillate flowers per inflorescence^1^**															**Average**
		**2**	**3**	**4**	**5**	**6**	**7**	**8**	**9**	**10**	**11**	**12**	**13**	**14**	**15**	**16**	
2012	Dawei	0^b^	0^b^	0.99^c^	5.94^c^	22.77^b^	10.89^bc^	20.79^b^	2.97^c^	17.82^b^	2.97^a^	5.94^b^	2.98^a^	0.99^a^	1.98^b^	2.97^a^	8.52^a^
	Bokehong	0^b^	0^b^	0.56^c^	6.36^c^	21.32^b^	9.92^c^	21.34^b^	4.41^b^	19.32^a^	1.67^b^	6.74^a^	3.21^a^	1.03^a^	2.01^b^	2.11^b^	8.54^a^
	Jinling	0^b^	0^b^	1.24^c^	5.11^d^	25.65^a^	11.32^ab^	20.54^b^	5.81^a^	13.76^c^	3.01^a^	7.21^a^	2.04^b^	0.45^c^	2.21^a^	1.65^c^	8.30^ab^
	Yuzhui	0^b^	0^b^	2.43^b^	8.10^b^	26.87^a^	12.26^a^	26.21^a^	3.11^c^	14.21^c^	1.76^b^	3.22^c^	1.07^c^	0.76^b^	0^c^	0^e^	7.60^b^
	*C.heterophylla*	1.01^a^	9.09^a^	27.27^a^	12.12^a^	15.15^c^	10.06^c^	10.14^c^	2.02^d^	7.07^d^	0^c^	0^d^	3.03^a^	0^d^	2.02^b^	1.01^d^	6.16^c^
2013	Dawei	0^b^	0^b^	1.24^c^	5.79^d^	20.37^b^	12.42^a^	21.37^a^	4.54^b^	15.39^b^	4.65^a^	5.54^b^	2.92^b^	2.16^a^	1.94^c^	1.67^c^	8.50^a^
	Bokehong	0^b^	0^b^	0.43^c^	6.53^c^	22.51^a^	9.53^c^	21.49^a^	3.99^c^	20.46^a^	2.07^d^	5.51^b^	3.80^a^	1.52^b^	1.07^d^	1.09^d^	8.41^a^
	Jinling	0^b^	0^b^	1.09^c^	6.24^cd^	23.99^a^	10.76^b^	19.34^b^	6.07^a^	14.03	3.28^b^	6.94^a^	1.91^c^	1.27^c^	2.37^a^	2.71^a^	8.45^a^
	Yuzhui	0^b^	0^b^	3.07^b^	8.27^b^	23.32^a^	11.34^b^	20.68^ab^	4.35^b^	15.47^b^	2.37^c^	3.33^c^	2.71^b^	0.94^d^	2.20^b^	1.95^b^	8.10^a^
	*C.heterophylla*	0.97^a^	9.47^a^	25.44^a^	10.29^a^	15.29^c^	12.89^a^	10.99^c^	2.14^d^	6.09^d^	1.37^e^	1.21^d^	1.97^c^	0^e^	0.94^d^	0.94^d^	5.88^b^

1 *Values are the percentage in each category of the total number of inflorescences sampled. The percentage values were arcsin-transformed before the analysis of variance. Values within the same column and year followed by different lowercase letters are significantly different at P ≤ 0.05 as indicated by LSD test*.

### Pistillate inflorescence and fruit cluster drop in open-pollinated and artificially pollinated flowers

Pistillate inflorescence drop ratio, fruit cluster drop ratio and total drop ratio in open-pollinated and artificially pollinated flowers were calculated in 2012 and 2013. Among open-pollinated inflorescences, the percentage of pistillate inflorescence drop varied from 36 to 54%, and the percentage of fruit cluster drop ranged from 4 to 27% (Table 2). Among the four hybrid hazel cultivars, no significant difference was observed in the percentage of total drop between open and artificial pollination. For *C. heterophylla*, the total drop after artificial pollination was significantly lower than those of the open-pollinated controls. Thus, artificial pollination significantly reduced total drop in *C. heterophylla*, but had no significant effect on total drop in the four hybrid hazel cultivars.

**Table 2:** **Pistillate inflorescence and fruit cluster drop after open (Control) and artificial pollination (AP) in four hybrid hazel cultivars and *C*. *heterophylla***.

**Cultivar/species**	**Treatment**	**2012**			**2013**		
		**Pistillate inflorescence drop (%)**	**Fruit cluster drop (%)**	**Total drop^1^ (%)**	**Pistillate inflorescence drop (%)**	**Fruit cluster drop (%)**	**Total drop (%)**
Dawei	Control	44.17 ± 2.25^bc^	19.30 ± 0.58^d^	63.47 ± 2.83^abc^	41.32 ± 1.69^bc^	18.96 ± 0.95^abc^	60.28 ± 2.64^a^
	AP	42.74 ± 1.86^bcd^	18.83 ± 0.69^d^	61.57 ± 2.55^abcd^	40.56 ± 1.85^bcd^	17.80 ± 0.83^bcd^	58.36 ± 2.68^ab^
Bokehong	Control	36.24 ± 1.45^f^	23.23 ± 1.01^b^	59.47 ± 2.47^cde^	39.87 ± 1.88^bcd^	17.60 ± 0.78^cd^	57.47 ± 2.66^ab^
	AP	37.10 ± 1.71^ef^	21.01 ± 0.98^c^	58.11 ± 2.69^de^	39.91 ± 1.64^bcd^	17.20 ± 0.77^d^	57.11 ± 2.41^ab^
Jinling	Control	40.07 ± 1.65^de^	26.07 ± 0.96^a^	66.14 ± 2.60^a^	42.38 ± 2.04^b^	17.79 ± 0.71^bcd^	60.17 ± 2.74^ab^
	AP	38.17 ± 1.52^ef^	26.57 ± 1.06^a^	64.74 ± 2.58^ab^	40.27 ± 2.00^bcd^	19.20 ± 0.96^ab^	59.47 ± 2.96^ab^
Yuzhui	Control	42.17 ± 1.30^bcd^	17.94 ± 0.82^d^	60.11 ± 2.12^cde^	38.24 ± 1.48^cd^	19.25 ± 0.84^a^	57.49 ± 2.32^ab^
	AP	45.67 ± 2.03^b^	13.77 ± 0.55^e^	59.44 ± 2.58^cde^	37.19 ± 1.58^d^	18.18 ± 0.90^bcd^	55.37 ± 2.48^b^
*C.heterophylla*	Control	53.47 ± 2.66^a^	9.31 ± 0.44^f^	62.78 ± 3.09^bcd^	50.97 ± 2.13^a^	4.67 ± 0.21^f^	55.64 ± 2.34^ab^
	AP	41.88 ± 1.32^cd^	13.36 ± 0.68^e^	55.24 ± 2.00^e^	40.37 ± 1.67^bcd^	9.90 ± 0.41^e^	50.27 ± 2.08^c^

1 *The sum of pistillate inflorescence and fruit cluster drop. Values within the same column followed by different lowercase letters are significantly different at P ≤ 0.05 as indicated by LSD test*.

### Fruit number per cluster after open and artificial pollination

Fruit number per cluster did not show a uniform or random distribution pattern (Table 3). A minimum of one fruit per cluster was recorded for all individuals. A maximum number of five to six fruit per cluster was recorded for “Dawei” and “Yuzhui” and six to seven for “Bokehong” and “Jinling” in the 2 years, respectively. For “Dawei” and “Yuzhui,” the percentage of clusters carrying more than three fruits per cluster was low, whereas the percentage of clusters with one to three fruits was comparatively higher. For “Bokehong” and “Jinling,” two to four were the most frequent numbers of fruits per cluster. Fruit clusters with three or four nuts showed the highest percentage frequency in *C. heterophylla*. The average fruit number per cluster of the four hybrid cultivars ranged from 2.1 to 3.1, which was significantly lower than that of *C. heterophylla*.

**Table 3:** **Distribution characteristics of fruit number per cluster after open (Control) and artificial pollination (AP) in four hybrid hazel cultivars and *C. heterophylla***.

**Year**	**Cultivar/species**	**Treatment**	**Number of fruit per cluster^1^**										**ANF**	**ANAO**	**PAO (%)**	**FS (%)**
			**1**	**2**	**3**	**4**	**5**	**6**	**7**	**8**	**9**	**10**				
2012	Dawei	Control	23.89 ± 1.03^b^	31.67 ± 1.39^b^	27.22 ± 1.24^c^	12.78 ± 0.58^f^	4.44 ± 0.21^e^	0 ± 0^g^	0 ± 0^e^	0 ± 0^c^	0 ± 0^b^	0 ± 0^b^	2.42 ± 0.11^de^	6.10 ± 0.28^a^	71.60 ± 3.41^a^	8.46 ± 0.41^f^
		AP	22.10 ± 0.88^c^	32.58 ± 1.60^b^	26.54 ± 1.15^cd^	14.41 ± 0.69^d^	4.37 ± 0.19^e^	0 ± 0^g^	0 ± 0^e^	0 ± 0^c^	0 ± 0^b^	0 ± 0^b^	2.46 ± 0.11^d^	6.06 ± 0.27^a^	71.13 ± 3.72^a^	9.02 ± 0.42^def^
	Bokehong	Control	1.64 ± 0.08^g^	32.79 ± 1.41^b^	35.24 ± 1.50^a^	26.23 ± 1.07^a^	3.28 ± 0.16^f^	0.82 ± 0.03^f^	0 ± 0^e^	0 ± 0^c^	0 ± 0^b^	0 ± 0^b^	2.99 ± 0.12^c^	5.55 ± 0.24^b^	64.96 ± 2.93^b^	9.77 ± 0.45^cde^
		AP	1.87 ± 0.06^g^	34.27 ± 1.10^b^	32.17 ± 1.21^b^	27.47 ± 1.16^a^	2.97 ± 0.13^f^	1.25 ± 0.06^e^	0 ± 0^e^	0 ± 0^c^	0 ± 0^b^	0 ± 0^b^	2.92 ± 0.13 ^c^	5.62 ± 0.25^b^	64.97 ± 3.00^b^	10.62 ± 0.47^c^
	Jinling	Control	10.53 ± 0.53^d^	31.58 ± 1.64^b^	34.21 ± 1.97^ab^	13.16 ± 0.64^ef^	5.26 ± 0.20^d^	2.63 ± 0.12^d^	2.63 ± 0.12^b^	0 ± 0^c^	0 ± 0^b^	0 ± 0^b^	2.89 ± 0.12^c^	5.41 ± 0.23 b	65.18 ± 3.02^b^	8.92 ± 0.40^def^
		AP	7.27 ± 0.32^e^	32.14 ± 1.57^b^	32.17 ± 1.56^b^	16.21 ± 0.66^c^	6.21 ± 0.26^c^	3.74 ± 0.14^c^	2.26 ± 0.10^c^	0 ± 0^c^	0 ± 0^b^	0 ± 0^b^	3.02 ± 0.14^c^	5.28 ± 0.21 b	63.61 ± 2.97 b	9.91 ± 0.46^cd^
	Yuzhui	Control	25.68 ± 0.82^a^	43.92 ± 1.36^a^	22.97 ± 0.91^e^	6.08 ± 0.23^h^	1.35 ± 0.05^g^	0 ± 0^g^	0 ± 0^e^	0 ± 0^c^	0 ± 0^b^	0 ± 0^b^	2.13 ± 0.08^f^	5.47 ± 0.22^b^	71.97 ± 3.23^a^	8.73 ± 0.42^ef^
		AP	22.39 ± 1.02^c^	45.92 ± 2.25^a^	21.74 ± 0.90^e^	8.24 ± 0.38^g^	1.71 ± 0.08^g^	0 ± 0^g^	0 ± 0^e^	0 ± 0^c^	0 ± 0^b^	0 ± 0^b^	2.20 ± 0.08^ef^	5.40 ± 0.22^b^	71.05 ± 3.34^a^	8.63 ± 0.42^ef^
	*C. heterophylla*	Control	10.71 ± 0.54^d^	8.93 ± 0.45^d^	35.71 ± 1.91^a^	19.64 ± 0.61^b^	12.50 ± 0.48^b^	8.93 ± 0.42^a^	1.79 ± 0.06^d^	1.79 ± 0.07^b^	0 ± 0^b^	0 ± 0^b^	3.57 ± 0.16^b^	2.59 ± 0.11 c	42.05 ± 1.97 c	20.26 ± 0.92^b^
		AP	4.28 ± 0.13^f^	15.39 ± 0.60^c^	24.27 ± 1.03^de^	15.59 ± 0.64^cd^	15.77 ± 0.65^a^	6.59 ± 0.24^b^	11.05 ± 0.52 ^a^	2.58 ± 0.11^a^	2.24 ± 0.10^a^	2.24 ± 0.11^a^	4.29 ± 0.19^a^	1.87 ± 0.08^d^	30.36 ± 1.44^d^	28.22 ± 1.31^a^
2013	Dawei	Control	21.42 ± 0.95^b^	33.67 ± 1.52^bc^	25.21 ± 1.22^d^	12.24 ± 0.55^e^	5.32 ± 0.27^d^	2.14 ± 0.08^d^	0 ± 0^f^	0 ± 0^c^	0 ± 0^a^	0 ± 0^a^	2.53 ± 0.11^d^	5.97 ± 0.26^a^	70.26 ± 3.27^abc^	9.21 ± 0.42^de^
		AP	23.87 ± 1.19^a^	35.22 ± 1.51^b^	21.49 ± 1.01^ef^	11.36 ± 0.53^ef^	3.24 ± 0.15^fg^	3.43 ± 0.14^c^	1.39 ± 0.06^d^	0 ± 0^c^	0 ± 0^a^	0 ± 0^a^	2.51 ± 0.11 ^d^	5.99 ± 0.27 ^a^	70.50 ± 3.12^ab^	9.47 ± 0.43^de^
	Bokehong	Control	1.07 ± 0.05^h^	35.49 ± 1.49^b^	36.42 ± 1.64^ab^	22.44 ± 0.90^c^	3.24 ± 0.15^fg^	1.34 ± 0.06^e^	0 ± 0^f^	0 ± 0^c^	0 ± 0^a^	0 ± 0^a^	2.95 ± 0.13^c^	5.46 ± 0.23^bc^	64.89 ± 3.00^cd^	10.39 ± 0.45^b^
		AP	0.92 ± 0.03^h^	31.32 ± 1.16^cd^	36.07 ± 1.53^ab^	27.18 ± 1.14^a^	3.07 ± 0.13^g^	1.44 ± 0.06 ^e^	0 ± 0^f^	0 ± 0^c^	0 ± 0^a^	0 ± 0^a^	3.04 ± 0.14^c^	5.37 ± 0.22^bc^	63.80 ± 2.95^d^	12.24 ± 0.55^c^
	Jinling	Control	8.25 ± 0.43^e^	32.89 ± 1.87^bc^	38.29 ± 1.68^a^	11.32 ± 0.55^ef^	4.82 ± 0.18^de^	2.46 ± 0.10^d^	1.97 ± 0.09^b^	0 ± 0^c^	0 ± 0^a^	0 ± 0^a^	2.87 ± 0.13 ^c^	5.58 ± 0.23^abc^	66.06 ± 3.12^bcd^	9.07 ± 0.41^e^
		AP	5.01 ± 0.25^f^	29.62 ± 1.42^d^	34.33 ± 1.52^b^	18.32 ± 0.75^d^	8.24 ± 0.33^c^	3.39 ± 0.14^c^	1.09 ± 0.05^e^	0 ± 0^c^	0 ± 0^a^	0 ± 0^a^	3.10 ± 0.14^c^	5.35 ± 0.21^c^	63.35 ± 2.94^d^	9.37 ± 0.43^de^
	Yuzhui	Control	21.07 ± 0.65^b^	45.66 ± 1.78^a^	19.42 ± 0.60^f^	9.23 ± 0.34^g^	4.62 ± 0.16^e^	0 ± 0^f^	0 ± 0^f^	0 ± 0^c^	0 ± 0^a^	0 ± 0^a^	2.31 ± 0.10 ^d^	5.79 ± 0.24^ab^	71.52 ± 3.14 ^a^	9.23 ± 0.42^de^
		AP	19.73 ± 0.98^c^	43.97 ± 1.80^a^	22.2 ± 0.95^e^	10.21 ± 0.47^fg^	3.89 ± 0.18^f^	0 ± 0^f^	0 ± 0^f^	0 ± 0^c^	0 ± 0^a^	0 ± 0^a^	2.35 ± 0.10^d^	5.75 ± 0.25^abc^	71.04 ± 3.30^ab^	9.52 ± 0.44^de^
	*C. heterophylla*	Control	15.31 ± 0.77^d^	7.24 ± 0.38^e^	30.86 ± 1.23^c^	21.26 ± 0.64^c^	13.71 ± 0.52 ^b^	7.16 ± 0.25^b^	3.47 ± 0.16^b^	0.99 ± 0.05^b^	0 ± 0^a^	0 ± 0^a^	3.51 ± 0.16^b^	2.37 ± 0.11^d^	40.28 ± 1.85^e^	22.17 ± 1.04^b^
		AP	3.27 ± 0.13^g^	9.66 ± 0.41^e^	25.94 ± 1.15^d^	24.9 ± 1.02^b^	15.61 ± 0.63^a^	10.32 ± 0.49^a^	8.33 ± 0.37^a^	1.97 ± 0.09^a^	0 ± 0^a^	0 ± 0^a^	4.14 ± 0.19^a^	1.74 ± 0.08^e^	29.58 ± 1.36^f^	32.64 ± 1.51^a^

1 *Values are the percentage in each category of the total number of clusters sampled. ANF, average number of fruit per cluster; ANAO, average number of abortive ovary per cluster; PAO, percentage of abortive ovaries per cluster; FS, fruit set as percentage of total number of pistillate flowers per plant. Values within the same column and year followed by different lowercase letters are significantly different at P ≤ 0.05 as indicated by LSD test*.

In open-pollinated inflorescences, the percentage abortive ovaries among the four hybrid hazel cultivars ranged from 63 to 72% and were significantly higher than that of *C. heterophylla*. Artificial pollination had no significant effect on ANF per cluster, ANAOs per cluster and PAO per cluster in each cultivar. However, artificial pollination significantly promoted ANF, whereas ANAO and PAO were significantly lowered in *C. heterophylla*. Thus, artificial pollination had no significant effect on the frequency of abortive ovaries in the hybrid hazel cultivars, but significantly reduced that in *C. heterophylla*.

In addition to the high frequency of pistillate inflorescence drop and young fruit drop in four hybrid hazel cultivars (58~67%), the development of most pollinated ovaries stopped soon after pollination. For instance, number of pistillate flowers per inflorescence and ANF per cluster was about 8.5 (Table 1) and 2.4 (Table 3) respectively in cultivar Dawei. Therefore, the percentage of pistillate flowers that set fruit per plant (FS) was pretty low. Among the four hybrid hazel cultivars, FS ranged from 8 to 12%. Artificial pollination had no significant effect on FS. FS in *C. heterophylla* was much higher than that of the four hybrid hazel cultivars. Artificial pollination significantly increased FS in *C. heterophylla* by promoting the proportion of pistillate flowers that set fruit. Thus, fruit drop was significantly reduced by artificial pollination in *C. heterophylla*.

### Nut mass characteristics, yield per cluster, and kernel ratio

Fruit fresh biomass characteristics of open-pollinated and artificially pollinated clusters carrying the same number of fruit were compared. Artificial pollination had no significant effect on nut biomass. Therefore, only nut biomass and related traits of open-pollinated clusters are shown in Table 4. The number of fruit per cluster increased, whereas shell mass, kernel mass and total nut mass tended to decrease. Thus, fruit number per cluster was inversely related to total nut mass of an individual fruit cluster. In contrast, the total yield per cluster was directly related to fruit number per cluster. Nut size was much smaller in *C. heterophylla* than those of the hybrid hazel cultivars. The total nut mass of the hybrid hazel cultivars was triple that of *C. heterophylla*. The kernel ratio of the four hybrid hazel cultivars ranged from 0.38 to 0.50. These results indicated that the fruit number per cluster was not related to the kernel ratio. The kernel ratio of *C. heterophylla* ranged from 0.30 to 0.39 and was slightly lower than those of the hybrid hazel cultivars.

**Table 4:** **Nut fresh biomass characteristics, yield per cluster and kernel ratio after open pollination (Control)**.

**Year**	**Cultivar/species**	**Fruit number per cluster**	**Shell mass (g)**	**Kernel mass (g)**	**Totalnut mass (g)**	**Fitting equation (*X*: Fruit number per cluster; *Y*: total nut mass)**	**Kernel ratio**	**Yield per cluster(g cluster^−1^)**	**Fitting equation (*X*: Fruit number per cluster; *Y*: Yield per cluster)**
2012	Dawei	1	2.78 ± 0.14^a^	1.68 ± 0.08^ab^	4.46 ± 0.21^a^	*Y* = −0.26X + 4.91	0.38 ± 0.00^a^	4.46 ± 0.21^d^	*Y* = 3.21X + 2.24
		2	2.68 ± 0.12^ab^	1.82 ± 0.08^a^	4.50 ± 0.20^a^		0.40 ± 0.00^a^	9.00 ± 0.39^c^	
		3	2.52 ± 0.11^bc^	1.72 ± 0.08^ab^	4.24 ± 0.18^ab^	*R*^2^ = 0.8067^*^	0.41 ± 0.00^a^	12.72 ± 0.55^b^	*R*^2^ = 0.9499^*^
		4	2.43 ± 0.10 ^c^	1.65 ± 0.07^b^	4.08 ± 0.17^b^		0.40 ± 0.00^a^	16.32 ± 0.68^a^	
		5	1.99 ± 0.09^d^	1.38 ± 0.06^c^	3.37 ± 0.15^c^		0.41 ± 0.00^a^	16.85 ± 0.74^a^	
	Bokehong	1	2.25 ± 0.11^b^	1.96 ± 0.09^a^	4.21 ± 0.20^ab^	*Y* = −0.464X + 5.0673	0.47 ± 0.00^a^	4.21 ± 0.20^e^	*Y* = 1.678X + 4.8253
		2	2.55 ± 0.11^a^	1.86 ± 0.08^a^	4.41 ± 0.19^a^		0.42 ± 0.00^bc^	8.82 ± 0.39^d^	
		3	2.33 ± 0.10^b^	1.68 ± 0.08^b^	4.01 ± 0.17^b^	*R*^2^ = 0.9015^*^	0.42 ± 0.00^bc^	12.03 ± 0.52^c^	*R*^2^ = 0.7637^*^
		4	1.90 ± 0.08^c^	1.38 ± 0.06^c^	3.28 ± 0.14^c^		0.42 ± 0.00^bc^	13.12 ± 0.55^ab^	
		5	1.46 ± 0.06^d^	1.03 ± 0.05^d^	2.49 ± 0.11^d^		0.41 ± 0.00^c^	12.45 ± 0.55^bc^	
		6	1.25 ± 0.05^e^	1.01 ± 0.05^d^	2.26 ± 0.10^d^		0.45 ± 0.00^ab^	13.56 ± 0.60^a^	
	Yuzhui	1	2.36 ± 0.12^a^	1.97 ± 0.09^a^	4.33 ± 0.21^a^	*Y* = −0.15X + 4.404	0.45 ± 0.00^bc^	4.33 ± 0.21^f^	*Y* = 3.54X + 0.942
		2	2.30 ± 0.10^a^	1.73 ± 0.08^bc^	4.03 ± 0.18^ab^		0.43 ± 0.00^c^	8.06 ± 0.35^e^	
		3	2.24 ± 0.09^a^	1.66 ± 0.07^bc^	3.90 ± 0.17^bc^	*R*^2^ = 0.9355^*^	0.43 ± 0.00^c^	11.70 ± 0.51^d^	*R*^2^ = 0.9988^*^
		4	2.24 ± 0.09^a^	1.59 ± 0.07^c^	3.83 ± 0.16^bc^		0.42 ± 0.00^c^	15.32 ± 0.64^c^	
		5	1.89 ± 0.08^b^	1.79 ± 0.08^b^	3.68 ± 0.16^cd^		0.49 ± 0.00^ab^	18.40 ± 0.81^b^	
	Jinling	1	2.16 ± 0.11^a^	1.70 ± 0.08^ab^	3.86 ± 0.18^a^	*Y* = −0.2546X + 4.17	0.44 ± 0.00^bc^	3.86 ± 0.18^e^	*Y* = 2.0761X + 3.2829
		2	1.81 ± 0.08^bc^	1.82 ± 0.08^a^	3.63 ± 0.16^ab^		0.50 ± 0.00^a^	7.26 ± 0.32^d^	
		3	1.87 ± 0.08^b^	1.58 ± 0.07^bc^	3.45 ± 0.15^b^	*R*^2^ = 0.9568^*^	0.46 ± 0.00^b^	10.35 ± 0.45^c^	*R*^2^ = 0.9105^*^
		4	1.70 ± 0.07^cd^	1.40 ± 0.06^d^	3.10 ± 0.13^c^		0.45 ± 0.00^b^	12.40 ± 0.52^b^	
		5	1.66 ± 0.07^cd^	1.47 ± 0.07^cd^	3.13 ± 0.14^c^		0.47 ± 0.00^ab^	15.65 ± 0.69 ^a^	
		6	1.56 ± 0.07^d^	1.08 ± 0.05^e^	2.64 ± 0.12^d^		0.41 ± 0.00^cd^	15.84 ± 0.70 ^a^	
		7	1.34 ± 0.07^e^	0.91 ± 0.04^f^	2.25 ± 0.11^e^		0.40 ± 0.00^d^	15.75 ± 0.76^a^	
	*C.heterophylla*	1	0.80 ± 0.04^e^	0.40 ± 0.02^e^	1.20 ± 0.06^e^	*Y* = −0.0273X + 1.7614	0.33 ± 0.00^bc^	1.20 ± 0.06^g^	*Y* = 1.3873X + 0.9886
		2	1.24 ± 0.05^b^	0.75 ± 0.03^a^	1.99 ± 0.09^a^		0.38 ± 0.00^a^	3.98 ± 0.17^f^	
		3	1.46 ± 0.06^a^	0.64 ± 0.03^b^	2.10 ± 0.09^a^	*R*^2^ = 0.053	0.30 ± 0.00^c^	6.30 ± 0.27^e^	*R*^2^ = 0.9625^*^
		4	1.03 ± 0.04^c^	0.64 ± 0.03^b^	1.67 ± 0.07^b^		0.38 ± 0.00^a^	6.68 ± 0.28^e^	
		5	1.03 ± 0.04^c^	0.58 ± 0.03^c^	1.61 ± 0.07^bc^		0.36 ± 0.00^ab^	8.05 ± 0.35^d^	
		6	1.06 ± 0.05^c^	0.54 ± 0.02^c^	1.60 ± 0.07^bcd^		0.34 ± 0.00^b^	9.60 ± 0.42^c^	
		7	0.90 ± 0.04^d^	0.58 ± 0.03^c^	1.48 ± 0.07^cd^		0.39 ± 0.00^a^	10.36 ± 0.50^b^	
		8	1.02 ± 0.04^c^	0.44 ± 0.02^d^	1.46 ± 0.06^d^		0.30 ± 0.00^c^	11.68 ± 0.51^a^	
2013	Dawei	1	2.83 ± 0.14^a^	1.71 ± 0.08^b^	4.54 ± 0.22^a^	*Y* = −0.3754X + 5.2107	0.38 ± 0.00^ab^	4.54 ± 0.22^e^	*Y* = 2.4097X + 4.1093
		2	2.72 ± 0.12^a^	1.89 ± 0.09^a^	4.61 ± 0.20^a^		0.41 ± 0.00^a^	9.22 ± 0.40^d^	
		3	2.64 ± 0.11^ab^	1.54 ± 0.07^c^	4.18 ± 0.18^b^	*R*^2^ = 0.9006^*^	0.37 ± 0.00^b^	12.54 ± 0.54^c^	*R*^2^ = 0.8552^*^
		4	2.46 ± 0.10^b^	1.50 ± 0.07^c^	3.96 ± 0.17^b^		0.38 ± 0.00^ab^	15.84 ± 0.66^b^	
		5	2.18 ± 0.09^c^	1.24 ± 0.06^d^	3.42 ± 0.15^c^		0.36 ± 0.00^bc^	17.10 ± 0.75^a^	
		6	1.78 ± 0.08^d^	0.89 ± 0.04^e^	2.67 ± 0.12^d^		0.33 ± 0.00^c^	16.02 ± 0.70^ab^	
	Bokehong	1	2.31 ± 0.12^b^	1.88 ± 0.08^a^	4.19 ± 0.20^b^	*Y* = −0.5023X + 5.1313	0.45 ± 0.00^a^	4.19 ± 0.20^d^	*Y* = 1.4923X + 5.1187
		2	2.67 ± 0.11^a^	1.92 ± 0.09^a^	4.59 ± 0.20^a^		0.42 ± 0.00^ab^	9.18 ± 0.40^c^	
		3	2.19 ± 0.09^b^	1.64 ± 0.07^b^	3.83 ± 0.17^c^	*R*^2^ = 0.9038^*^	0.43 ± 0.00^ab^	11.49 ± 0.50^b^	*R*^2^ = 0.7316^*^
		4	1.80 ± 0.07^c^	1.24 ± 0.06^c^	3.04 ± 0.13^d^		0.41 ± 0.00^b^	12.16 ± 0.51^ab^	
		5	1.46 ± 0.06^d^	1.05 ± 0.05^d^	2.51 ± 0.11^e^		0.42 ± 0.00^ab^	12.55 ± 0.55^a^	
		6	1.21 ± 0.05^e^	0.87 ± 0.04^e^	2.08 ± 0.09^f^		0.42 ± 0.00^ab^	12.48 ± 0.55^a^	
	Yuzhui	1	2.58 ± 0.13^a^	1.94 ± 0.09^a^	4.52 ± 0.22^a^	*Y* = −0.28X + 4.8543	0.43 ± 0.00^cd^	4.52 ± 0.22^f^	*Y* = 2.555X + 3.5971
		2	2.44 ± 0.10^a^	1.77 ± 0.08^b^	4.21 ± 0.18^ab^		0.42 ± 0.00^cd^	8.42 ± 0.37^e^	
		3	2.41 ± 0.10^ab^	1.68 ± 0.08^bc^	4.09 ± 0.18^bc^	*R*^2^ = 0.9806^*^	0.41 ± 0.00^d^	12.27 ± 0.53^d^	*R*^2^ = 0.94^*^
		4	2.23 ± 0.09^b^	1.61 ± 0.07^cd^	3.84 ± 0.16^cd^		0.42 ± 0.00^cd^	15.36 ± 0.64^c^	
		5	1.93 ± 0.08^c^	1.58 ± 0.07^cd^	3.51 ± 0.15^de^		0.45 ± 0.00^bc^	17.55 ± 0.77^b^	
		6	1.69 ± 0.07^d^	1.50 ± 0.07^de^	3.19 ± 0.14^e^		0.47 ± 0.00^ab^	19.14 ± 0.84^a^	
		7	1.42 ± 0.07^e^	1.36 ± 0.06^e^	2.78 ± 0.13^f^		0.49 ± 0.00^a^	19.46 ± 0.93^a^	
	Jinling	1	2.16 ± 0.11^a^	1.63 ± 0.07^ab^	3.79 ± 0.18^a^	*Y* = −0.219X + 3.955	0.43 ± 0.00^bc^	3.79 ± 0.18^e^	*Y* = 2.686X + 1.398
		2	1.80 ± 0.08^b^	1.72 ± 0.08^a^	3.52 ± 0.15^a^		0.49 ± 0.00^a^	7.04 ± 0.31^d^	
		3	1.69 ± 0.07^b^	1.50 ± 0.07^b^	3.19 ± 0.14^b^	*R*^2^ = 0.9633	0.47 ± 0.00^ab^	9.57 ± 0.42^c^	*R*^2^ = 0.9966
		4	1.75 ± 0.07^b^	1.32 ± 0.06^c^	3.07 ± 0.13^b^		0.43 ± 0.00^bc^	12.28 ± 0.52^b^	
		5	1.75 ± 0.08^b^	1.17 ± 0.05^d^	2.92 ± 0.13^b^		0.40 ± 0.00^c^	14.60 ± 0.64^a^	
	*C.heterophylla*	1	0.85 ± 0.04^e^	0.44 ± 0.02^e^	1.29 ± 0.06^e^	*Y* = −0.0487X + 1.7704	0.34 ± 0.00^cd^	1.29 ± 0.06^h^	*Y* = 1.2457X + 1.1193
		2	1.26 ± 0.05^a^	0.81 ± 0.04^a^	2.07 ± 0.09^a^		0.39 ± 0.00^a^	4.14 ± 0.18^g^	
		3	1.15 ± 0.05^b^	0.59 ± 0.03^b^	1.74 ± 0.07^b^	*R*^2^ = 0.2143	0.34 ± 0.00^cd^	5.22 ± 0.22^f^	*R*^2^ = 0.9671^*^
		4	1.02 ± 0.04^c^	0.57 ± 0.03^bc^	1.59 ± 0.07^c^		0.36 ± 0.00^abc^	6.36 ± 0.26^e^	
		5	1.00 ± 0.04^c^	0.54 ± 0.02^cd^	1.54 ± 0.07^c^		0.35 ± 0.00^bcd^	7.70 ± 0.34^d^	
		6	0.94 ± 0.04^cd^	0.55 ± 0.02^bcd^	1.49 ± 0.07^cd^		0.37 ± 0.00^abc^	8.94 ± 0.39^c^	
		7	0.86 ± 0.04^de^	0.51 ± 0.02^d^	1.37 ± 0.07^de^		0.38 ± 0.00^ab^	9.59 ± 0.46^b^	
		8	0.90 ± 0.04^de^	0.42 ± 0.02^e^	1.32 ± 0.06^e^		0.32 ± 0.00^d^	10.56 ± 0.46^a^	

* *Indicates a significant difference at the P ≤ 0.05 level. Values within the same column and year for each cultivar/species followed by different lowercase letters are significantly different at P ≤ 0.05 as indicated by LSD test*.

### External morphology and pollen tube growth in developing and abortive ovaries during progamic phase

**Figure 1:**
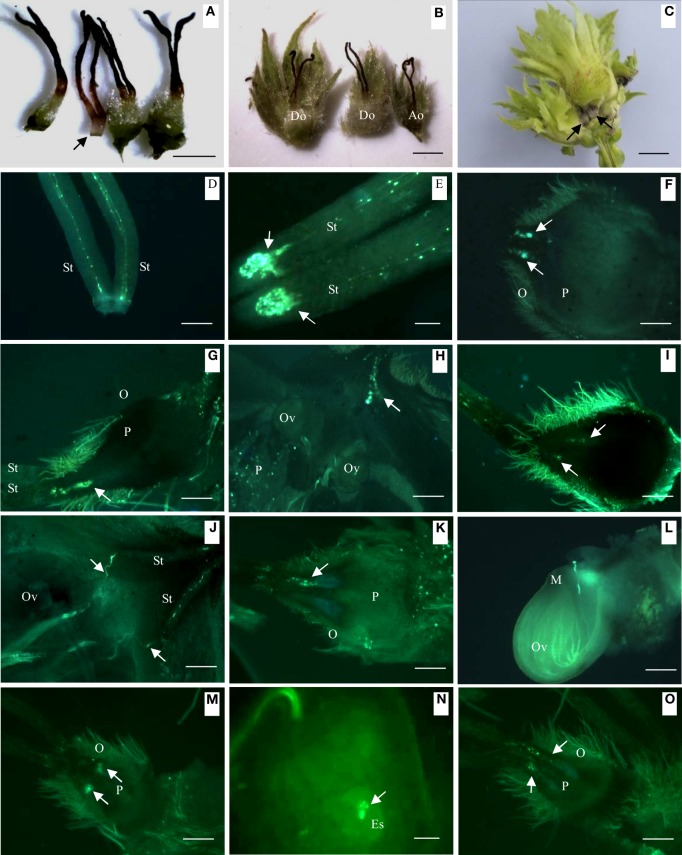
**Differences in external morphology and pollen tube growth in developing and abortive ovaries during the progamic phase**. **(A)** External morphology of abortive ovary (indicated by arrow) and developing ovary, 30 days after blooming. **(B)** Normally developing ovary (left and center) and abortive ovary (right), 40 days after blooming. **(C)** Normally developing fruit and abortive fruit in a fruit cluster, 65 days after pollination. Arrows indicate abortive ovaries. **(D)** Young pollen tubes in the style, 5 days after blooming. **(E)** Pollen tubes curled and arrested at the style base, showed by arrows, 20 days after blooming. **(F)** Pollen tubes growing into developing ovary, arrows showed pollen tubes, 40 days after blooming. **(G)** Pollen tubes (showed by arrow) growing into abortive ovary, 40 days after pollination. **(H)** Pollen tubes (showed by arrow) growing toward ovule, 45 days after pollination. **(I)** Pollen tubes arrested in abortive ovary (showed by arrow), 45 days after pollination. **(J)** Pollen tubes in style (showed by arrow) changed direction and grew toward ovule, 50 days after pollination. **(K)** Pollen tubes arrested in abortive ovary, 50 days after pollination. **(L)** Pollen tubes starting to penetrate ovule of developing ovary, 55 days after pollination. **(M)** Abortive ovary with Curled and arrested pollen tubes, 55 days after pollination. **(N)** Pollen tubes in developing ovary release two sperm cells in embryo sac, 60 days after blooming. **(O)** Abortive ovary with curled and arrested pollen tubes, 60 days after blooming. Key: Ao, abortive ovary; Do, developing ovary; Es, embryo sacs; M, micropyle; O, ovary; Ov, ovule; P, parenchyma; St, style; Scale bars: **A,B** = 1 mm; **C** = 400 μm; **D** = 300 μm; **E** = 100 μm; **F–O** = 300 μm.

About 30–40 days after blooming, the ovary visibly began to develop. At this stage, abortive ovaries were easily distinguished from developing ovaries (Figures 1A,B). The diameter of abortive ovaries remained less than 2 mm during the entire inflorescence and fruit development stages, which was much smaller than that of developing ovaries. About 60–70 days after blooming, abortive ovaries began to harden and wither (Figure 1C), whereas developing ovaries within the same fruit cluster continued to grow until harvest.

Soon after blooming and pollination, pollen grains germinated on the stigma. The pollen tubes grew to the style base within a few days (Figure 1D). Because no obvious ovary had developed at anthesis, the pollen tubes curled at the style base and awaited development of the ovary and ovules (Figure 1E) for about 20 days. About 40 days after blooming, pollen tubes maintain curly in the style (Figure 1F). Although smaller in diameter than developing ovaries, pollen tubes in abortive ovaries were observed to reach a similar location as in developing ovaries 40 days after blooming (Figure 1G). Two ovules were observed in a developing ovary by fluorescence microscopy 45 days after blooming. At this stage, pollen tubes in the style began to grow toward the developing ovule (Figure 1H). During the same stage, in the abortive ovary pollen tube growth was completely arrested at the style base until ovary withering and thus never grew beyond the style base (Figures 1I,K,M,O). A single pollen tube was observed to change direction and grew to the ovule in the developing ovary on 50th day after blooming (Figure 1J). This pollen tube passed though vicinity of the micropyle and stretched forward without entering the micropyle (Figure 1L). Finally, the pollen tube entered the embryo sac to release two sperm cells (Figure 1N) on the 60th day after blooming.

### Anatomy of abortive and developing ovaries during progamic phase

**Figure 2:**
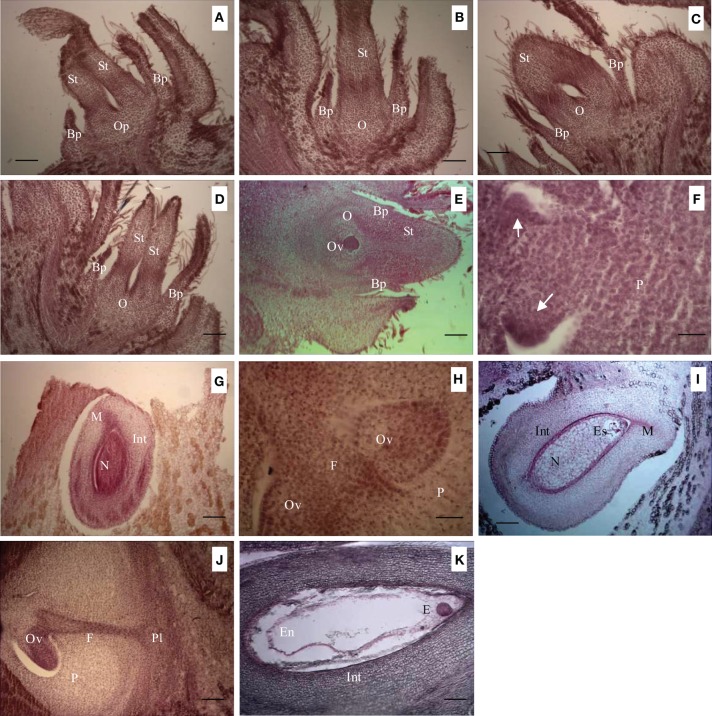
**Anatomical differences in abortive and young developing fruit during the progamic phase**. **(A)** Early ovary primordium, 20 days after blooming. **(B)** Early rudimentary ovary, 25 days after blooming. **(C)** Developing ovary, 30 days after blooming. **(D)** Abortive ovary, 30 days after blooming. **(E)** Developing ovary with early ovule, 40 days after blooming. **(F)** Abortive ovary with early ovule primordia indicated by arrows, 40 days after blooming. **(G)** Ovule in developing ovary, 50 days after blooming. **(H)** Ovules in abortive ovary, 50 days after blooming. **(I)** Ovule with mature embryo sac in developing ovary, 55 days after blooming. **(J)** Ovule lacking differentiated embryo sac and nucellus in abortive ovary, 55 days after blooming. **(K)** Embryo in fertilized ovule of developing ovary, 65 days after blooming. Key: Bp, bract primordium; E, embryo; En, endosperm; Es, embryo sac; F, funiculus; Int, integument; M, micropyle; N, nucellus; Op, ovary primordium; O, ovary; Ov, ovule; P, parenchyma; Pl, placenta; St, style. Scale bars: **A,B** = 100 μm; **C–E** = 100 μm; **F** = 30 μm; **G** = 100 μm; **H** = 30 μm; **I–K** = 100 μm.

The ovary began to develop gradually after blooming. About 20 days after booming, an early ovary primordium was obvious (Figure 2A). The ovary primordium developed and differentiated further and an early rudimentary ovary had formed on about the 25th day after blooming (Figure 2B). The difference in diameter of developing and abortive ovaries was apparent on about the 30th day after blooming (Figures 2C,D). During this stage, ovules were not discernible in either ovary type (Figures 2C,D). In the developing ovary, a spherical ovule had developed in the approximate center of the ovary (Figure 2E) by about the 40th day after blooming. At the same stage, only a hemispherical ovule primordium had formed in the abortive ovary (Figure 2F). In the developing ovary, the ovule began to differentiate an integument and the embryo sac began to develop in the nucellus by the 50th day after blooming (Figure 2G). In the abortive ovary, an oval ovule had formed at the same stage but its differentiation had not begun (Figure 2H). A mature embryo sac in the developing ovary was observed on the 55th day after blooming (Figure 2I). No embryo sac was observed in the abortive ovary prior to it withering (Figure 2J). Soon after mature embryo sac formation, a spherical embryo was observed on about the 65th day after blooming (Figure 2K). In brief, the development of abortive ovaries was much slower than that of normally developing ovaries. The diameter of abortive ovaries was maintained at 1–2.0 mm during its life cycle. Although an ovule developed to a certain degree in the abortive ovary, a mature ovule containing an embryo sac never developed. Subsequently, abortive ovaries began to dehydrate and wither. Thus, it was impossible for the ovule in an abortive ovary to be fertilized.

### Comparison of starch content in abortive and developing ovaries during the progamic phase

Starch content in abortive and developing ovaries during the progamic phase was measured to clarify the relationship between ovary abortion and starch accumulation, and the results were showed in Table 5. In the year of 2013–2014, for the developing ovary, its starch content decreased from 30 to 50 days after blooming, while starch content in abortive ovary fluctuated within the specific limits. Compared with abortive ovary, starch content in developing ovary of four hybrid hazel cultivars and *C. heterophylla* were significantly higher without exception.

**Table 5:** **Starch content in ovaries of four hybrid hazel cultivars and *C. heterophylla***.

**Cultivar/species**	**Treatment**	**2013**			**2014**		
		**30 DAB (μg·g^−1^ FW)**	**40 DAB (μg·g^−1^ FW)**	**50 DAB (μg·g^−1^ FW)**	**30 DAB (μg·g^−1^ FW)**	**40 DAB (μg·g^−1^ FW)**	**50 DAB (μg·g^−1^ FW)**
Dawei	DO	490.4 ± 22.71^bc^	427.9 ± 19.18^a^	350.4 ± 17.01^b^	510.2 ± 2.34^a^	452.6 ± 20.16^a^	339.1 ± 14.71^bc^
	AO	217.7 ± 10.38 ^d^	243.1 ± 11.32^c^	200.4 ± 9.33^e^	247.0 ± 11.67^d^	201.7 ± 9.52^de^	240.4 ± 11.24^e^
Bokehong	DO	512.3 ± 21.16^ab^	451.6 ± 18.45^a^	371.2 ± 15.49^ab^	480.3 ± 19.47^ab^	401.5 ± 15.69^b^	359.8 ± 13.64^ab^
	AO	221.8 ± 8.05^d^	231.4 ± 8.22^cd^	196.4 ± 7.12^e^	231.2 ± 8.34^de^	193.4 ± 7.12^e^	185.0 ± 6.42^f^
Jinling	DO	479.1 ± 20.75^c^	390.4 ± 16.77^b^	314.3 ± 13.74^c^	450.2 ± 19.19^bc^	352.1 ± 15.35^c^	321.4 ± 13.82^c^
	AO	214.2 ± 9.21^d^	211.5 ± 9.32^de^	230.4 ± 9.22^d^	232.1 ± 9.90^de^	193.2 ± 8.24^e^	201.2 ± 8.56^f^
Yuzhui	DO	534.7 ± 20.32^a^	430.5 ± 16.01^a^	390.1 ± 14.97^a^	504.1 ± 19.06^a^	441.8 ± 16.77^a^	376.1 ± 14.39^a^
	AO	195.0 ± 8.25^d^	201.4 ± 8.69^e^	214.3 ± 9.09^de^	207.2 ± 8.86^ef^	222.3 ± 9.44^d^	184.5 ± 7.89^f^
*C.heterophylla*	DO	479.1 ± 22.82^c^	390.4 ± 19.04^b^	290.3 ± 13.85^c^	443.9 ± 20.15^c^	378.4 ± 18.34^bc^	278.1 ± 13.45^d^
	AO	223.8 ± 11.03^d^	196.5 ± 10.07^e^	199.0 ± 9.61^e^	195.6 ± 9.38^f^	206.8 ± 10.40^de^	190.3 ± 9.07^f^

*DO and AO indicate developing ovary and abortive ovary respectively and DAB represents days after blooming. Values within the same column followed by different lowercase letters are significantly different at P ≤ 0.05*.

## Discussion

### Relationship between fruit number per cluster and fruit size

Fruit size is dependent mostly on cell number rather than cell size because cell number is the only factor determining sink strength (Bertin et al., 2002; Corelli-Grappadelli and Lakso, 2004). Mature fruit size is positively correlated with flower size and, particularly, ovary size at blooming (Rosati et al., 2009). During fruit development, fruit growth is mostly achieved through cell expansion (Rosati et al., 2012). Among several olive cultivars with different fruit sizes, genetic differences in fruit size appear to arise from differences in cell division patterns in the ovary (and probably in the whole flower) before blooming. The final fruit size, aside from genetic control, is also related to environmental and endogenous plant conditions that allow the genetic potential for growth to be achieved to a varying degree. European hazel (*Corylus avellana* L.) is a species with a peculiar floral biology characteristic. Petals, ovaries and ovules are absent in pistillate inflorescences at anthesis. Thus, the final nut size is not determined by flower size or cell number in the ovary at anthesis as is generally true. Compared with *C. heterophylla*, the nuts produced by the four hybrid hazel cultivars in the present study showed a much larger mass and size (Table 4), suggesting that genetic control plays an important role in determining the final nut size. In addition, fruit number per cluster was positively correlated with yield per cluster and thus was indicated to be a key factor determining sink strength in hazel (Table 4). Fruit number per cluster is always negatively correlated with nut mass (Table 4), implying limited sink strength had to sustain a greater number of fruit in a cluster and resulted in smaller nuts. Thus, a higher number of fruit per cluster was beneficial to increase the total yield but at the expense of a reduction in individual fruit size.

### Possible causal factors for ovary abortion in hazel

Many causes trigger the abortion of reproductive structures, including resource limitation (Herrera, 1991), pollen limitation (Burd, 1994), interaction between resource and pollen limitation (Juenger and Bergelson, 1997), damage caused by biological agents (Parker, 1987), adverse climatic conditions (Lee and Bazzaz, 1982) and the genetic background of the plant. Ovary abortion in olive appears to be related with resource competition among flowers; conditions that affect competition among flowers/fruits or that decrease available resources usually result in increased abortion (Reale et al., 2009). Pistil abortion is also known to be under genetic control and varies among cultivars (Lavee et al., 2002). Even genetic control can be explained with the competition theory (Rosati et al., 2011). Large-fruited cultivars tend to show a higher frequency of pistil abortion compared with small-fruited cultivars (Rosati et al., 2011). Compared with the small-fruited *C. heterophylla*, the PAO per cluster in the four large-fruited hybrid hazel cultivars was significantly higher (Table 3). Furthermore, the ANF per cluster was not promoted by artificial pollination in the hybrid hazel cultivars. Thus, a higher frequency of ovary abortion and an invariable ANF per cluster in the four hybrid cultivars might be associated with insufficient available resources for all flowers to develop. Competition within pistillate inflorescence might have led to early ovary abortion, which compensated for the limited resources. In *C. heterophylla*, artificial pollination significantly reduced the loss of yield caused by pistillate inflorescence drop and fruit drop (Table 2). In addition, the PAO and ANF per cluster of *C. heterophylla* were reduced and promoted by artificial pollination, respectively (Table 3). These results indicated that fruit set was at least partly determined by pollen availability.

The reproductive success of *Opuntia microdasys* is simultaneously limited by the availability of nutrients and compatible pollen, the effect of florivory, and genetic incompatibility (Piña et al., 2007). In hazel, incompatibility is determined sporophytically and is controlled by a single locus with multiple alleles (Ma et al., 2013). Mixed pollen of five individuals was used in artificial supplementary pollinations to avoid incompatibility between the pollen and stigma in the present study. Fluorescence microscopic results indicated that pollen grains germinated well on the stigma and pollen tubes grew to the style base at early developmental stages of both developing and abortive ovaries. Subsequently, pollen tubes in the abortive ovary remained curled near the style base in the ovary and were unable to further and fertilize the ovule. A pollen tube in the developing ovary was observed to change direction and grow to the ovule on the 50th day after blooming. Thereafter, the pollen tube entered the integument and the embryo sac of the ovule on the 55th and 60th days after blooming, respectively. Thus, pollen incompatibility was not the factor leading to ovary abortion in hazel. Histological examination indicated that the ovule integument and embryo sac failed to develop in the abortive ovary prior to it withering. Deficiency of starch in flower of olive (*Olea europaea* L.) and within the ovule of *apricot* (*Prunus armeniaca* L.) might be involved in determining their abortion (Rodrigo and Herrero, 1998; Reale et al., 2006). Starch reserves in the ovary of avocado could play a significant role in the reproductive process (Alcaraz et al., 2010). Starch content in developing ovaries of four hybrid hazel cultivars and *C. heterophylla* was significantly higher than that in abortive ovary (Table 5), and the result was in accordance with that the reports mentioned above. Thus, the inability of pollen tubes to penetrate the ovule, the absence of a mature embryo sac in the ovule and deficiency starch were three important factors that led to ovary abortion. Nutrient addition increased seed production and decreased ovary abortion in *Ipomopsis aggregata* (Campbell and Halama, 1993). Whether spraying with nutrient solution before fertilization could reduce ovary abortion and increase final yield at harvest requires further research.

## Conclusions

Besides loss due to pistillate inflorescence and fruit cluster drop, the development of most of the pistillates flower will stop and form abortive ovaries, and this process occurs during progamic stage of hazelnut. There are 5.8–8.5 pistillate flowers per pistillate inflorescence in four hybrid hazel cultivars and *C. heterophylla* (Table 1). At harvest, their fruit number per cluster ranged from 2.4 to 4.3 (Table 2). The PAO in the four hybrid hazel cultivars ranged from 63 to 72%, and was significantly higher than that of *C. heterophylla* (29–42%) (Table 3). The results of artificial pollination treatment indicated that only the abortive ovary ratio of *C. heterophylla* was significantly reduced following artificial pollination, and fruit number per cluster was positively and negatively correlated with yield and nut mass, respectively. Anatomy results of abortive ovary indicated that an integument seldom differentiated and a mature embryo sac never developed (Figure 1). Pollen tube growth of abortive ovaries showed that their pollen tube growth was arrested at the style base and was incapable to fertilize the ovules (Figure 2). Thus, fertilization of their ovules was precluded. Compared with abortive ovary, starch content in developing ovary of five tested germplasms were significantly higher. To conclude, abortive ovary was incapable to finish fertilization process due to the absence of mature embryo sac and arrested pollen tubes. For the four hybrid cultivars, abortive ovary was likely associated with insufficient resource availability to support fruit set by all flowers, whereas ovary abortion in *C. heterophylla* was at least partly determined by pollen availability.

### Conflict of interest statement

The authors declare that the research was conducted in the absence of any commercial or financial relationships that could be construed as a potential conflict of interest.
